# Bis(1*H*-benzimidazole-κ*N* ^3^)bis­[2-(naphthalen-1-yl)acetato-κ^2^ *O*,*O*′]manganese(II) monohydrate

**DOI:** 10.1107/S1600536812007441

**Published:** 2012-02-24

**Authors:** Zhen-Ming Zhang, Fu-Jun Yin, Shu-An Li, Li-Ping Wang

**Affiliations:** a College of Environment Science and Spatial Infomatics, China University of Mining & Technology, Xuzhou, Jiangsu 221008, People’s Republic of China; b Department of Chemical Engineering, Huaihai Institute of Technology, Lianyungang 222005, People’s Republic of China; c Jiangsu Marine Resources Development Research Institute, Huaihai Institute of Technology, Lianyungang 222005, People’s Republic of China

## Abstract

In the title compound, [Mn(C_12_H_9_O_2_)_2_(C_7_H_6_N_2_)_2_]·H_2_O, the Mn^II^ ion is located on a twofold rotation axis and six-coordinated, displaying a distorted MnN_2_O_4_ octa­hedral geometry. The crystal packing is stabilized by N—H⋯O hydrogen bonds, which give rise to a one-dimensional structure along [001], and π–π inter­actions between the imidazole rings and between the benzene rings of the 2-(naphthalen-1-yl)acetate ligands [centroid–centroid distances = 3.761 (3) and 3.728 (4) Å]. The contribution of the electron density associated with the disordered water molecules was not considerd in the final structure model.

## Related literature

For related structures with 2-(naphthalen-1-yl)acetate ligands, see: Duan *et al.* (2007 ▶); Ji *et al.* (2011 ▶); Tang *et al.* (2006 ▶); Yang *et al.* (2008 ▶); Yin *et al.* (2011 ▶).
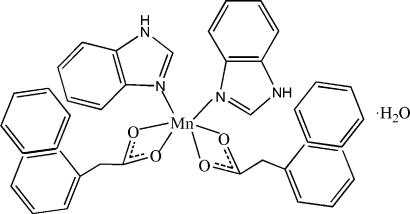


## Experimental

### 

#### Crystal data

[Mn(C_12_H_9_O_2_)_2_(C_7_H_6_N_2_)_2_]·H_2_O
*M* *_r_* = 679.62
Monoclinic, 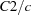

*a* = 11.654 (7) Å
*b* = 20.013 (12) Å
*c* = 14.329 (12) Å
β = 106.148 (7)°
*V* = 3210 (4) Å^3^
*Z* = 4
Mo *K*α radiation
μ = 0.46 mm^−1^
*T* = 298 K
0.10 × 0.10 × 0.10 mm

#### Data collection

Bruker APEXII CCD diffractometer
Absorption correction: multi-scan (*SADABS*; Sheldrick, 1996 ▶) *T*_min_ = 0.955, *T*_max_ = 0.955
12089 measured reflections
2830 independent reflections
1819 reflections with *I* > 2σ(*I*)
*R*_int_ = 0.068

#### Refinement

*R*[*F* ^2^ > 2σ(*F* ^2^)] = 0.044
*wR*(*F* ^2^) = 0.090
*S* = 1.00
2830 reflections
213 parameters
H-atom parameters constrained
Δρ_max_ = 0.21 e Å^−3^
Δρ_min_ = −0.19 e Å^−3^

### 

Data collection: *APEX2* (Bruker, 2007 ▶); cell refinement: *SAINT* (Bruker, 2007 ▶); data reduction: *SAINT*; program(s) used to solve structure: *SHELXS97* (Sheldrick, 2008 ▶); program(s) used to refine structure: *SHELXL97* (Sheldrick, 2008 ▶); molecular graphics: *XP* in *SHELXTL* (Sheldrick, 2008 ▶) and *DIAMOND* (Brandenburg, 1999 ▶); software used to prepare material for publication: *SHELXTL* and *PLATON* (Spek, 2009 ▶).

## Supplementary Material

Crystal structure: contains datablock(s) I, global. DOI: 10.1107/S1600536812007441/hy2515sup1.cif

Structure factors: contains datablock(s) I. DOI: 10.1107/S1600536812007441/hy2515Isup2.hkl

Additional supplementary materials:  crystallographic information; 3D view; checkCIF report

## Figures and Tables

**Table 1:** Hydrogen-bond geometry (Å, °)

*D*—H⋯*A*	*D*—H	H⋯*A*	*D*⋯*A*	*D*—H⋯*A*
N2—H2⋯O2^i^	0.86	1.99	2.791 (4)	154

Symmetry code: (i) 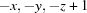.

**Fig. 1.**
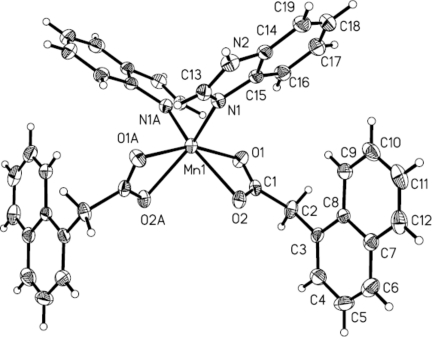
Molecular structure of the title compound. Displacement ellipsoids are drawn at the 30% probability level. [Symmetry code: (A) -x, y, 3/2-z.]

**Fig. 2.**
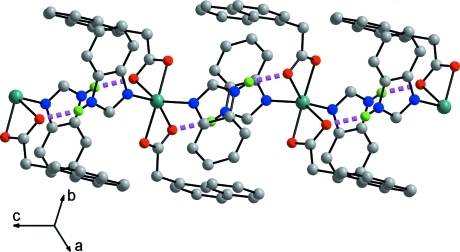
Part of the one-dimensional structure of the title compound, formed by N—H···O hydrogen bonds (dashed lines). H atoms not involved in hydrogen bonding are omitted for clarity.

## supplementary crystallographic information

### Comment

In recent years many interests have been focused on 2-(naphthalen-1-yl)acetate ligand in coordination chemistry due to its ability to form metal complexes (Duan *et al.*, 2007; Ji *et al.*, 2011; Tang *et al.*, 2006; Yang *et al.*, 2008; Yin *et al.*, 2011). The crystal structure of the title compound was determined as part of an ongoing study of the properties of manganese complexes containing benzimidazole ligands.

In the title mononuclear complex (Fig. 1), the Mn^II^ ion is located on a twofold rotation axis and six-coordinated by two N-donor atoms from two benzimidazoles and four O-donor atoms from two 2-(naphthalen-1-yl)acetate anions, displaying a distorted MnN_2_O_4_ octahedral geometry, with Mn—O bond lengths of 2.181 (2) and 2.339 (2) Å and a Mn—N bond length of 2.153 (2) Å. The solvent water molecules could not be modeled as discrete atomic sites. The crystal packing is stabilized by intermolecular N—H···O hydrogen bonds (Table 1), which give rise to a one-dimensional structure (Fig. 2). π–π ineractions between the imidazole rings and between the benzene rings of the 2-(naphthalen-1-yl)acetate ligands [centroid–centroid distances = 3.761 (3) and 3.728 (4) Å] are observed. An analogue cadmium(II) complex has been reported previously (Duan *et al.*, 2007).

### Experimental

The title compound was synthesized by the reaction of Mn(NO_3_)_2_.4H_2_O (75 mg, 0.3 mmol), 1-naphthylacetic acid (93 mg, 0.5 mmol), benzimidazole (35.4 mg, 0.3 mmol) and NaOH (20 mg, 0.5 mmol) in 16 ml of water/ethanol (v/v 2:1) under solvothermal conditions. The mixture was homogenized and transferred into a sealed Teflon-lined solvothermal bomb (volume: 25 ml) and heated to 160°C for three days. After cooling, colorless crystals of the title compound were obtained, which were washed with distilled water and absolute ethanol (yield: 46.3% based on Mn).

### Refinement

H atoms were placed in calculated positions and refined as riding atoms, with N—H = 0.86, C—H = 0.93 (CH) and 0.97 (CH_2_) Å and with *U*_iso_(H) = 1.2*U*_eq_(C, N). The structure contains one disordered solvent water molecules, which could not be modeled as discrete atomic sites. We employed the SQUEEZE subroutine in PLATON (Spek, 2009) to remove the water molecules.

### Crystal data

**Table:**

[Mn(C_12_H_9_O_2_)_2_(C_7_H_6_N_2_)_2_]·H_2_O	*F*(000) = 1372
*M**_r_* = 679.62	*D*_x_ = 1.369 Mg m^−^^3^
Monoclinic, *C*2/*c*	Mo *K*α radiation, λ = 0.71073 Å
Hall symbol: -C 2yc	Cell parameters from 1147 reflections
*a* = 11.654 (7) Å	θ = 2.3–18.0°
*b* = 20.013 (12) Å	µ = 0.46 mm^−^^1^
*c* = 14.329 (12) Å	*T* = 298 K
β = 106.148 (7)°	Block, colorless
*V* = 3210 (4) Å^3^	0.10 × 0.10 × 0.10 mm
*Z* = 4	

### Data collection

**Table:**

Bruker APEXII CCD diffractometer	2830 independent reflections
Radiation source: fine-focus sealed tube	1819 reflections with *I* > 2σ(*I*)
Graphite monochromator	*R*_int_ = 0.068
φ and ω scans	θ_max_ = 25.0°, θ_min_ = 2.1°
Absorption correction: multi-scan (*SADABS*; Sheldrick, 1996)	*h* = −13→13
*T*_min_ = 0.955, *T*_max_ = 0.955	*k* = −23→23
12089 measured reflections	*l* = −16→16

### Refinement

**Table:**

Refinement on *F*^2^	Primary atom site location: structure-invariant direct methods
Least-squares matrix: full	Secondary atom site location: difference Fourier map
*R*[*F*^2^ > 2σ(*F*^2^)] = 0.044	Hydrogen site location: inferred from neighbouring sites
*wR*(*F*^2^) = 0.090	H-atom parameters constrained
*S* = 1.00	*w* = 1/[σ^2^(*F*_o_^2^) + (0.0307*P*)^2^] where *P* = (*F*_o_^2^ + 2*F*_c_^2^)/3
2830 reflections	(Δ/σ)_max_ < 0.001
213 parameters	Δρ_max_ = 0.21 e Å^−^^3^
0 restraints	Δρ_min_ = −0.19 e Å^−^^3^

### Special details

**Table:**

Geometry. All esds (except the esd in the dihedral angle between two l.s. planes) are estimated using the full covariance matrix. The cell esds are taken into account individually in the estimation of esds in distances, angles and torsion angles; correlations between esds in cell parameters are only used when they are defined by crystal symmetry. An approximate (isotropic) treatment of cell esds is used for estimating esds involving l.s. planes.
Refinement. Refinement of F^2^ against ALL reflections. The weighted R-factor wR and goodness of fit S are based on F^2^, conventional R-factors R are based on F, with F set to zero for negative F^2^. The threshold expression of F^2^ > 2sigma(F^2^) is used only for calculating R-factors(gt) etc. and is not relevant to the choice of reflections for refinement. R-factors based on F^2^ are statistically about twice as large as those based on F, and R- factors based on ALL data will be even larger.

### Fractional atomic coordinates and isotropic or equivalent isotropic displacement parameters (Å^2^)

**Table:**

	*x*	*y*	*z*	*U*_iso_*/*U*_eq_	
Mn1	0.0000	0.02104 (3)	0.7500	0.0408 (2)	
O1	0.18427 (16)	0.04197 (10)	0.83166 (13)	0.0572 (5)	
O2	0.11161 (15)	0.10711 (9)	0.70743 (12)	0.0494 (5)	
N1	0.01727 (19)	−0.04724 (10)	0.63856 (15)	0.0417 (6)	
N2	−0.0295 (2)	−0.09629 (11)	0.49442 (15)	0.0504 (6)	
H2	−0.0711	−0.1080	0.4373	0.060*	
C1	0.1968 (2)	0.08917 (15)	0.7784 (2)	0.0432 (7)	
C2	0.3144 (2)	0.12562 (14)	0.80207 (19)	0.0523 (8)	
H2A	0.3178	0.1557	0.8559	0.063*	
H2B	0.3780	0.0931	0.8240	0.063*	
C3	0.3389 (2)	0.16536 (14)	0.72073 (19)	0.0436 (7)	
C4	0.3436 (2)	0.23303 (15)	0.7247 (2)	0.0584 (8)	
H4	0.3303	0.2549	0.7780	0.070*	
C5	0.3681 (3)	0.27052 (16)	0.6501 (3)	0.0698 (10)	
H5	0.3716	0.3169	0.6549	0.084*	
C6	0.3868 (3)	0.24044 (18)	0.5716 (3)	0.0679 (9)	
H6	0.4023	0.2661	0.5224	0.082*	
C7	0.3829 (2)	0.17035 (16)	0.5635 (2)	0.0511 (7)	
C8	0.3589 (2)	0.13228 (14)	0.63879 (19)	0.0412 (7)	
C9	0.3563 (2)	0.06224 (15)	0.6286 (2)	0.0532 (8)	
H9	0.3427	0.0359	0.6779	0.064*	
C10	0.3731 (2)	0.03232 (17)	0.5483 (3)	0.0661 (9)	
H10	0.3701	−0.0140	0.5431	0.079*	
C11	0.3948 (3)	0.0703 (2)	0.4742 (3)	0.0746 (11)	
H11	0.4061	0.0494	0.4195	0.090*	
C12	0.3995 (3)	0.1376 (2)	0.4811 (2)	0.0671 (9)	
H12	0.4139	0.1626	0.4308	0.081*	
C13	−0.0650 (2)	−0.05600 (13)	0.5549 (2)	0.0460 (7)	
H13	−0.1400	−0.0361	0.5399	0.055*	
C14	0.0854 (3)	−0.11580 (13)	0.5397 (2)	0.0449 (7)	
C15	0.1141 (2)	−0.08492 (12)	0.63008 (19)	0.0409 (7)	
C16	0.2253 (3)	−0.09474 (14)	0.6959 (2)	0.0536 (8)	
H16	0.2454	−0.0749	0.7570	0.064*	
C17	0.3040 (3)	−0.13490 (16)	0.6668 (3)	0.0680 (9)	
H17	0.3795	−0.1421	0.7090	0.082*	
C18	0.2742 (3)	−0.16529 (16)	0.5757 (3)	0.0733 (10)	
H18	0.3302	−0.1922	0.5586	0.088*	
C19	0.1649 (3)	−0.15643 (14)	0.5110 (2)	0.0618 (9)	
H19	0.1448	−0.1769	0.4504	0.074*	

### Atomic displacement parameters (Å^2^)

**Table:**

	*U*^11^	*U*^22^	*U*^33^	*U*^12^	*U*^13^	*U*^23^
Mn1	0.0346 (3)	0.0473 (4)	0.0410 (4)	0.000	0.0109 (3)	0.000
O1	0.0497 (13)	0.0629 (14)	0.0558 (13)	−0.0104 (10)	0.0093 (10)	0.0105 (11)
O2	0.0330 (11)	0.0661 (14)	0.0464 (11)	−0.0017 (9)	0.0065 (9)	0.0026 (10)
N1	0.0402 (14)	0.0450 (14)	0.0393 (13)	0.0013 (11)	0.0101 (11)	0.0001 (11)
N2	0.0587 (17)	0.0528 (16)	0.0371 (13)	−0.0058 (13)	0.0092 (12)	−0.0024 (12)
C1	0.0345 (17)	0.056 (2)	0.0409 (17)	−0.0010 (14)	0.0136 (14)	−0.0100 (15)
C2	0.0361 (16)	0.074 (2)	0.0443 (17)	−0.0125 (15)	0.0076 (13)	−0.0035 (15)
C3	0.0327 (16)	0.0474 (19)	0.0503 (17)	−0.0071 (13)	0.0106 (13)	−0.0003 (15)
C4	0.0475 (19)	0.055 (2)	0.069 (2)	−0.0020 (16)	0.0099 (16)	−0.0101 (18)
C5	0.063 (2)	0.043 (2)	0.094 (3)	−0.0040 (17)	0.007 (2)	0.007 (2)
C6	0.055 (2)	0.072 (3)	0.073 (2)	−0.0055 (18)	0.0113 (18)	0.021 (2)
C7	0.0359 (17)	0.059 (2)	0.0562 (19)	0.0024 (15)	0.0081 (14)	0.0076 (17)
C8	0.0298 (15)	0.0433 (18)	0.0484 (17)	0.0018 (13)	0.0075 (13)	0.0033 (14)
C9	0.0405 (18)	0.052 (2)	0.066 (2)	0.0010 (15)	0.0128 (15)	−0.0006 (16)
C10	0.0413 (19)	0.065 (2)	0.085 (3)	0.0085 (17)	0.0065 (18)	−0.022 (2)
C11	0.045 (2)	0.111 (3)	0.066 (2)	0.017 (2)	0.0111 (18)	−0.018 (2)
C12	0.044 (2)	0.104 (3)	0.052 (2)	0.0104 (19)	0.0117 (16)	0.009 (2)
C13	0.0428 (18)	0.0490 (18)	0.0462 (18)	−0.0002 (14)	0.0124 (15)	0.0027 (14)
C14	0.0521 (19)	0.0386 (17)	0.0484 (18)	−0.0001 (14)	0.0216 (15)	0.0021 (14)
C15	0.0464 (18)	0.0352 (16)	0.0436 (16)	0.0002 (13)	0.0166 (14)	0.0024 (13)
C16	0.050 (2)	0.0483 (19)	0.0581 (19)	0.0027 (15)	0.0084 (16)	0.0032 (15)
C17	0.049 (2)	0.059 (2)	0.094 (3)	0.0127 (17)	0.0150 (19)	0.006 (2)
C18	0.071 (3)	0.054 (2)	0.107 (3)	0.0116 (19)	0.047 (2)	0.003 (2)
C19	0.081 (3)	0.049 (2)	0.066 (2)	−0.0016 (18)	0.038 (2)	−0.0037 (16)

### Geometric parameters (Å, º)

**Table:**

Mn1—N1	2.153 (2)	C7—C12	1.411 (4)
Mn1—O1	2.181 (2)	C7—C8	1.411 (4)
Mn1—O2	2.339 (2)	C8—C9	1.409 (4)
O1—C1	1.248 (3)	C9—C10	1.359 (4)
O2—C1	1.260 (3)	C9—H9	0.9300
N1—C13	1.322 (3)	C10—C11	1.385 (4)
N1—C15	1.391 (3)	C10—H10	0.9300
N2—C13	1.331 (3)	C11—C12	1.350 (4)
N2—C14	1.372 (3)	C11—H11	0.9300
N2—H2	0.8600	C12—H12	0.9300
C1—C2	1.505 (4)	C13—H13	0.9300
C2—C3	1.503 (4)	C14—C19	1.378 (4)
C2—H2A	0.9700	C14—C15	1.390 (3)
C2—H2B	0.9700	C15—C16	1.389 (4)
C3—C4	1.356 (4)	C16—C17	1.369 (4)
C3—C8	1.423 (4)	C16—H16	0.9300
C4—C5	1.398 (4)	C17—C18	1.394 (4)
C4—H4	0.9300	C17—H17	0.9300
C5—C6	1.346 (4)	C18—C19	1.362 (4)
C5—H5	0.9300	C18—H18	0.9300
C6—C7	1.407 (4)	C19—H19	0.9300
C6—H6	0.9300		
			
N1^i^—Mn1—N1	101.22 (12)	C8—C3—C2	120.3 (2)
N1^i^—Mn1—O1	90.30 (8)	C3—C4—C5	121.2 (3)
N1—Mn1—O1	103.80 (8)	C3—C4—H4	119.4
N1^i^—Mn1—O1^i^	103.80 (8)	C5—C4—H4	119.4
N1—Mn1—O1^i^	90.30 (8)	C6—C5—C4	120.9 (3)
O1—Mn1—O1^i^	157.86 (11)	C6—C5—H5	119.6
N1^i^—Mn1—O2	146.52 (7)	C4—C5—H5	119.6
N1—Mn1—O2	95.77 (8)	C5—C6—C7	120.4 (3)
O1—Mn1—O2	57.50 (7)	C5—C6—H6	119.8
O1^i^—Mn1—O2	104.75 (8)	C7—C6—H6	119.8
N1^i^—Mn1—O2^i^	95.77 (8)	C6—C7—C12	121.6 (3)
N1—Mn1—O2^i^	146.52 (7)	C6—C7—C8	118.9 (3)
O1—Mn1—O2^i^	104.75 (8)	C12—C7—C8	119.4 (3)
O1^i^—Mn1—O2^i^	57.50 (7)	C9—C8—C7	117.5 (3)
O2—Mn1—O2^i^	85.16 (10)	C9—C8—C3	123.0 (3)
N1^i^—Mn1—C1^i^	100.87 (8)	C7—C8—C3	119.5 (3)
N1—Mn1—C1^i^	118.47 (9)	C10—C9—C8	121.5 (3)
O1—Mn1—C1^i^	132.61 (10)	C10—C9—H9	119.3
O1^i^—Mn1—C1^i^	28.56 (7)	C8—C9—H9	119.3
O2—Mn1—C1^i^	95.93 (9)	C9—C10—C11	120.5 (3)
O2^i^—Mn1—C1^i^	28.94 (7)	C9—C10—H10	119.7
N1^i^—Mn1—C1	118.47 (9)	C11—C10—H10	119.7
N1—Mn1—C1	100.86 (8)	C12—C11—C10	120.2 (3)
O1—Mn1—C1	28.56 (7)	C12—C11—H11	119.9
O1^i^—Mn1—C1	132.61 (10)	C10—C11—H11	119.9
O2—Mn1—C1	28.94 (7)	C11—C12—C7	120.8 (3)
O2^i^—Mn1—C1	95.93 (9)	C11—C12—H12	119.6
C1^i^—Mn1—C1	116.79 (13)	C7—C12—H12	119.6
C1—O1—Mn1	94.76 (16)	N1—C13—N2	113.1 (3)
C1—O2—Mn1	87.15 (17)	N1—C13—H13	123.4
C13—N1—C15	104.5 (2)	N2—C13—H13	123.4
C13—N1—Mn1	124.24 (19)	N2—C14—C19	132.5 (3)
C15—N1—Mn1	130.88 (17)	N2—C14—C15	105.3 (2)
C13—N2—C14	107.7 (2)	C19—C14—C15	122.1 (3)
C13—N2—H2	126.2	C16—C15—C14	120.5 (3)
C14—N2—H2	126.2	C16—C15—N1	130.1 (3)
O1—C1—O2	120.6 (2)	C14—C15—N1	109.4 (2)
O1—C1—C2	118.9 (2)	C17—C16—C15	117.0 (3)
O2—C1—C2	120.5 (3)	C17—C16—H16	121.5
O1—C1—Mn1	56.68 (13)	C15—C16—H16	121.5
O2—C1—Mn1	63.91 (14)	C16—C17—C18	121.9 (3)
C2—C1—Mn1	175.6 (2)	C16—C17—H17	119.0
C3—C2—C1	116.1 (2)	C18—C17—H17	119.0
C3—C2—H2A	108.3	C19—C18—C17	121.5 (3)
C1—C2—H2A	108.3	C19—C18—H18	119.3
C3—C2—H2B	108.3	C17—C18—H18	119.3
C1—C2—H2B	108.3	C18—C19—C14	117.0 (3)
H2A—C2—H2B	107.4	C18—C19—H19	121.5
C4—C3—C8	119.0 (3)	C14—C19—H19	121.5
C4—C3—C2	120.7 (3)		

Symmetry code: (i) −*x*, *y*, −*z*+3/2.

### Hydrogen-bond geometry (Å, º)

**Table:**

*D*—H···*A*	*D*—H	H···*A*	*D*···*A*	*D*—H···*A*
N2—H2···O2^ii^	0.86	1.99	2.791 (4)	154

Symmetry code: (ii) −*x*, −*y*, −*z*+1.
